# Highlight selection of radiochemistry and radiopharmacy developments by editorial board

**DOI:** 10.1186/s41181-026-00505-4

**Published:** 2026-10-07

**Authors:** Sarah Spreckelmeyer, Jean DaSilva, Clemens Decristoforo, Robert H. Mach, Jan Passchier, Giuseppe Carlucci, Adriano Duatti, Bart T. Cornelissen, Antonia Denkova, Jeroen Hendrikx, Yann Seimbille, Xing Yang, Hongmei Jia, Ming-Rong Zhang, Lars Perk, Peter Caravan, Jan Rijn Zeevaart, Julien Leenhardt, Juan Pellico, Mohammed Hussain Al-Qahtani, Zhen Cheng, Tamer Sakr, Min Yang

**Affiliations:** 1 Department of Nuclear Medicine, Charité – Universitätsmedizin Berlin, corporate member of Freie Universität Berlin and Humboldt-Universität zu Berlin, Augustenburger Platz 1, 13353 Berlin, Germany; 2 Université de Montréal, Montreal, Canada; 3 Innsbruck Medical University, Innsbruck, Austria; 4 University of Pennsylvania, Philadelphia, USA; 5 CITRAS Global Ltd., London, UK; 6 Jubilant Radiopharma, Kirkland, Canada; 7 University of Ferrara, Ferrara, Italy; 8 University Medical Center Groningen, Groningen, The Netherlands; 9 Delft University of Technology, Delft, The Netherlands; 10 The Netherlands Cancer Institute, Amsterdam, The Netherlands; 11 Erasmus Medical Center, Rotterdam, The Netherlands; 12 Peking University First Hospital, Beijing, China; 13 Beijing Normal University, Beijing, China; 14 National Institutes for Quantum Science and Technology, Chiba, Japan; 15 Radboud University Medical Center, Nijmegen, The Netherlands; 16 Massachusetts General Hospital, Boston, USA; 17 NECSA, NuMeRI NPC, University of Pretoria, Pretoria, Pretoria, Pretoria, South Africa; 18 Université Grenoble Alpes, Saint-Martin-d’Hères, France; 19 Institut de Ciència de Materials de Barcelona, Cerdanyola des Vallès, Barcelona, Spain; 20 Cyclotron & Radiopharmaceutical Department, King Faisal Specialist Hospital & Research Centre, Riyadh, Saudi Arabia; 21 Shanghai Institute of Materia Medica, Chinese Academy of Sciences, Beijing, Pudong China; 22 Egyptian Atomic Energy Authority, Cairo, Egypt; 23 Jiangsu Institute of Nuclear Medicine, Wuxi, China

**Keywords:** Highlight articles, Radiochemistry, Radiopharmacy, Radiopharmaceutical sciences, Nuclear medicine

## Abstract

**Background:**

The Editorial Board of EJNMMI Radiopharmacy and Chemistry releases a biannual highlight commentary to update the readership on trends in the field of radiopharmaceutical development and application of radiopharmaceuticals.

**Main Body:**

This selection of highlights provides commentary on 23 different topics selected by each co-authoring Editorial Board member addressing a variety of aspects ranging from novel radiochemistry, proof-of-concepts, preclinical and early clinical studies using novel radiopharmaceuticals.

**Conclusion:**

Trends in radiochemistry and radiopharmacy are highlighted. Hot topics cover the entire scope of EJNMMI Radiopharmacy and Chemistry, demonstrating the progress in the research field in many aspects.

## Background

Each individual co-authoring member of the Editorial Board has selected to highlight an article that has appeared in the radiochemistry, radiopharmacy and imaging agent literature during the period January-June 2026. The aim of this collaborative initiative is to create a biyearly overview for the readers summarizing the latest trends and hot topics in the field.

## Extension of the chelator toolbox to porphyrins for “CopperNostics” (e.g. ^64^Cu,^67^Cu)

By Sarah Spreckelmeyer

The highlighted paper “Preferential Formation and Stability of Copper Porphyrins amidst other Metals in Dilute Aqueous Solution—Applications for Radiopharmaceuticals” (Cao et al. 2026) systematically investigates why copper inserts into porphyrin macrocycles more readily and forms more persistent complexes than many competing biologically relevant metal ions under mild aqueous conditions. Using a series of seven structurally distinct porphyrins, the authors compare metalation kinetics and product stability in water at room temperature, conditions that are directly relevant to radiopharmaceutical preparation.

This work addresses one of the central challenges in radiochemistry: maintaining kinetic and thermodynamic stability of radiometal complexes in vivo.

Over the past decade, the radiopharmaceutical community has increasingly shifted from simply maximizing thermodynamic stability constants toward optimizing kinetic inertness, since even highly stable complexes may undergo slow trans-chelation to proteins such as albumin or superoxide dismutase in vivo. Copper radiopharmaceuticals are particularly sensitive to this issue because Cu(II) readily participates in biological metal trafficking pathways. Reviews over the last few years have emphasized that improved chelator design remains one of the major priorities for clinical copper radiopharmaceutical development.

Against this background, the present study is significant because it demonstrates that porphyrins themselves possess an intrinsic chemical preference for copper.

This has several implications:

- It provides a fundamental coordination chemistry rationale for why copper–porphyrin systems can be robust radiopharmaceutical platforms.
- It suggests that porphyrins may serve not only as biological targeting or photodynamic therapy agents but also as highly stable copper chelators.

The work supports further development of ^64^Cu-labeled porphyrins for PET imaging and ^67^Cu analogues for targeted radionuclide therapy, particularly as theranostic pairs.

## Enhanced hydrophilicity translates into higher imaging contrast in FAP tumors

By Jean N. DaSilva

Introducing an *N*-methyl-benzenesulfonic acid side chain onto the FAPI-46 scaffold to increase hydrophilicity provided a radioligand labelled *via* [^18^F]AlF-NOTA chelation displaying rapid urinary and low hepatobiliary excretion, which is favourable for PET imaging abdominal fibroblast activation protein (FAP)-rich tumours/metastases. Such increase in polarity did not compromise binding characteristics as [^18^F]AlF-NOTA-GL01 exhibits higher FAP binding affinity (IC_50_ = 2.94 nM) and metabolic stability than [^18^F]FAPI-42 (Mu et al. 2026). Cell uptake and preclinical PET studies demonstrated high cell internalization and tumour uptake that remained stable for more than two hours in vivo. High tumour accumulation was blocked with FAPI-42 confirming binding specificity to FAP. Tumour uptake in FAP-negative A549 xenograft mice was lower than in A549-hFAP tumours. In patients with confirmed solid tumours, [^18^F]AlF-NOTA-GL01 was predominantly excreted via the urinary system, while tumour uptake was still high up to 120 min post-injection and displayed higher target-to-background ratios than both [^18^F]FDG and [^18^F]FAPI-42.

This paper shows that using a structure-guided design approach, the authors developed a more hydrophilic ^18^F-labeled derivative of the clinically used [^68^Ga]FAPI-46 in higher batch activity that combines higher affinity, high binding specificity and stability, renal clearance, low hepatobiliary background, and high lesion detectability with excellent imaging contrast in FAP-targeted primary and metastatic sites.

## Treatment monitoring of bacterial infections with PET using a novel targeted synthetic siderophore

By Clemens Decristoforo

**Fig. 1:**
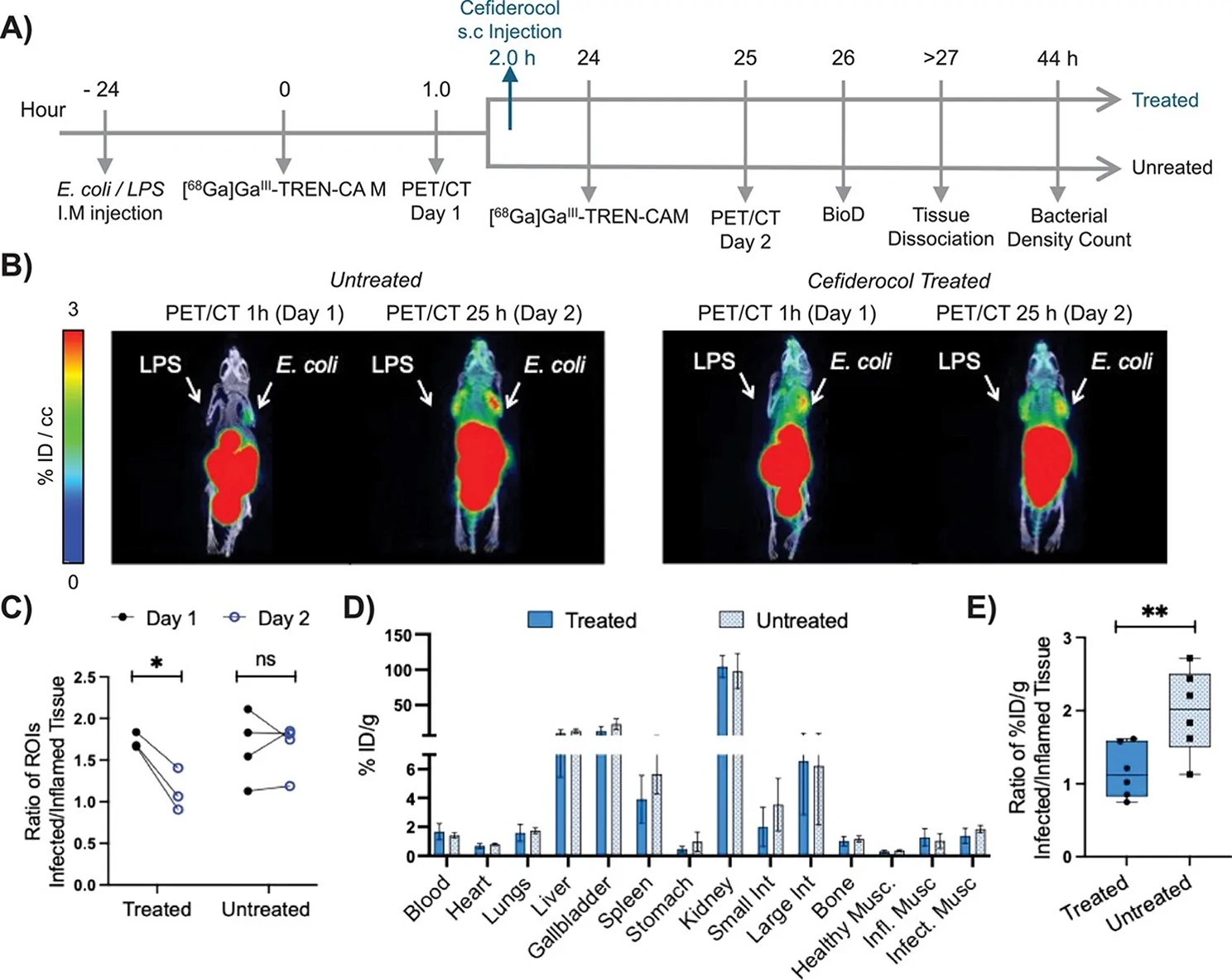
Use of [^68^Ga]GaIII-TREN‐CAM to track the progress of an E. coli K12 infection in BALB/c mice upon treatment with cefiderocol. (**A**) Descriptive timeline of the animal study. (**B**) Representative positron emission tomography/computed tomography images of [^68^Ga]Ga^III^‐TREN‐CAM showing the progress of the E. coli K12 infection in specimens untreated or treated with cefiderocol. Images are collected prior to administration of cefiderocol treatment (or saline control) and 24 h post administration of treatment (or saline vehicle) and are normalized to units of %ID/cc and presented as maximum intensity projection scans (MIPS). Activity is shown in the infected triceps (E. coli) and inflamed triceps (LPS). (**C**) Correlation plot showing the infected muscle‐to‐inflamed muscle ROI ratios for specimens of the treated and untreated cohort before (day 1) and after administration of treatment or saline vehicle (day 2), respectively. Statistical differences were calculated using two‐tailed Student’s t tests assuming unequal variances; * *p* = 0.0418. Region of interest (ROI) values correspond to the quantified percentage of injected dose per cubic centimeter of organ from the PET/CT images. (**D**) Ex vivo biodistribution profile of [^68^Ga]GaIII‐TREN‐CAM 2 h post injection on day 2 after assessment of antibiotic treatment via PET/CT Imaging. Error bars represent ± 1 standard deviation (*n* = 6). % ID/g of organ is the percentage of injected dose per gram of organ weight. (**E**) Comparison of the infected muscle‐to‐inflamed muscle %ID/g ratios for treated (*n* = 6) and untreated (*n* = 6) cohort before and after treatment. Statistical differences were calculated using two‐tailed Student’s t tests assuming unequal variances; ** *p* = 0.0181 (from Joaqui-Joaqui et al. 2026)

Current imaging approaches for bacterial infections suffer from the lack of microbe-specific radiopharmaceuticals. Several recent developments, primarily those targeting bacterial specific metabolism, hold promise to impact clinical research and patient management (Signore et al. 2023), also in view of increasing development of resistance and new antibiotic strategies evolving. One promising group are siderophores, low molecular weight iron chelators that are well-known virulence factors for pathogenic bacteria, targeting the iron metabolism of microbes (Dvorakova Bendova et al. 2025). Their use for PET imaging is enabled simply by replacing iron by ^68^Ga. In a recent article in *Angewandte Chemie* (Joaqui-Joaqui et al. 2026), the group of Eszter Boros characterized a synthetic Enterobactin analogue (TREN-CAM). They evaluated its recognition by bacteria, its uptake properties in comparison with [^67/68^Ga]Ga^III^-deferoxamine (DFO) and [^67/68^Ga]Ga^III^-enterobactin as well as their in vivo targeting of infections including therapy monitoring using a novel antibiotic with siderophore mediated mechanism (cefiderocol). They could provide convincing data on the specific and highly efficient uptake of [^68^Ga]Ga^III^-TREN-CAM, better than [^68^Ga]Ga^III^-DFO for some gram-negative bacteria, and showed improved selective targeting of infection in vivo over [^68^Ga]Ga^III^-enterobactin. [^68^Ga]Ga^III^-TREN-CAM also enabled imaging of treatment response after cefiderocol treatment (see Fig. 1).

Even though the concept of using ^68^Ga-labelled siderophores is not novel as such, in this paper the authors showed the suitability of a synthetic siderophore for PET imaging of bacterial infection and for monitoring novel antibiotic treatments, highlighting the potential of this concept for radiopharmaceutical development. The publication in a very high-ranking chemistry journal also indicates the high interest of the general research community in developing targeted imaging agents beyond the field of oncology.

## Late-stage radiofluorination using triarylselenonium triflates

By Robert H. Mach

Fluorine-18 continues to be the “radionuclide of choice” for many PET imaging studies, particularly in CNS imaging studies. The direct attachment of ^18^F to an aromatic ring was initially problematic and required the nucleophilic displacement of a leaving group that was activated by the presence of an electron-withdrawing group, preferably in the *para*-position of the ring. Radiosynthesis often required a multi-step process leading to the radiotracer being obtained in moderate to low radiochemical yield. The development of “late-stage” fluorination methods in the early 2000s represented a significant breakthrough in PET radiochemistry, since ^18^F could be introduced into structurally complex molecules in one or two synthetic steps (with the second step usually being deprotection of an amine or hydroxy group). Examples of this methodology include the use of aryl iodonium salts, iodonium ylides, boronic acids/esters, and stannanes. A limitation of these methods is the use of a transition metal such as copper, palladium, or nickel, which can complicate HPLC purification of the radiotracer and downstream quality assurance analyses, depending on residual metal concentration and toxicity. In this regard, a late-stage transition metal-free radiofluorination method offers clear PET production advantages.

**Fig. 2:**
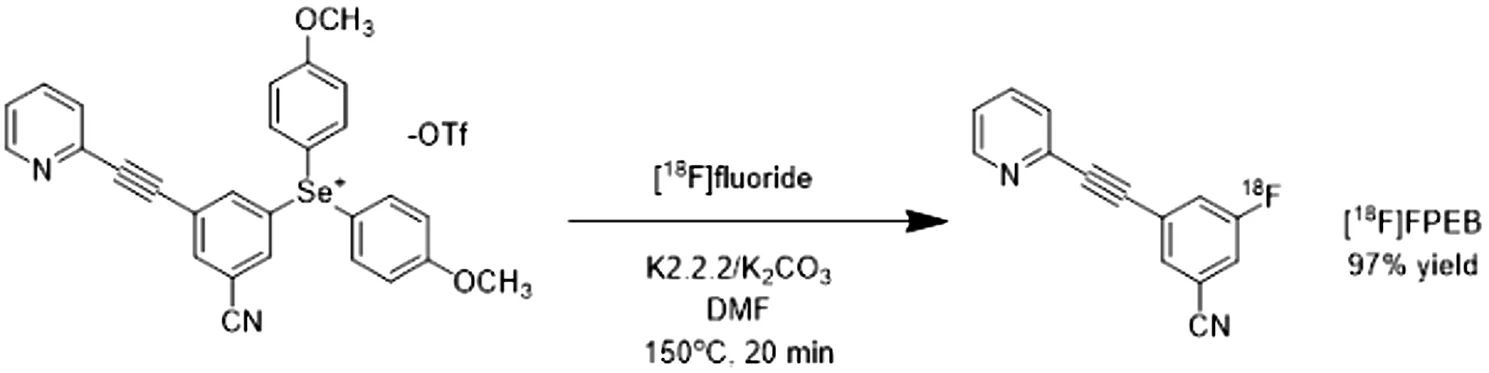
Reaction scheme for late-stage ^18^F-fluorination using triarylselenonium triflates

A recent article describes the use of triarylselenonium triflates for late-stage radiofluorination without transition metals (Sypniewski et al. 2026). This paper extended prior work on the use of triarylsulfonium salts as precursors for ^18^F-radiosynthesis, noting that selenium is more electrophilic and has greater leaving-group capacity than sulphur. The authors point out that there are a variety of synthetic routes available for making triarylselenonium salts, although many still require multi-step protocols. The authors initially started with a panel of asymmetric, monosubstituted triarylselenonium triflates to determine potential substituent effects on the ^18^F-incorporation. As is often the case, a higher yield was observed when the aromatic ring had an electron-withdrawing substituent, but modest yields were observed with precursors having a mild electron-donating substituent (i.e., an alkyl group). An exception was the corresponding *para*-F-substrate, which gave a surprisingly low radiochemical yield of [^18^F]1,4-difluorobenzene, driven by electronic preference for the phenyl rings and possibly by deleterious fluorine exchange. Radiochemical yields were generally higher for the triarylselenonium triflates versus the corresponding triphenylsulfonium triflates when a head-to-head comparison was made. Modest to high yields were also obtained with *meta*- and *ortho*-substituted substrates, but the article could benefit from a more comprehensive comparison using more varied substitution patterns. Radiochemical yields tended to be high when ^18^F was introduced via thermal reactions (150 °C/20 min) and protocols using microwave irradiation also gave high labelling efficiency. Since the incorporation yield was very low with a *para*-methoxy substituent, the authors used the asymmetric aryl(di-*p*-anisyl)selenonium triflate to bias reactivity toward the target aryl fragment. This was showcased in the radiosynthesis of two PET radiotracer derivatives: the mGluR5 receptor radiotracer, [^18^F]FPEB (Fig. 2), and an ^18^F-labeled canagliflozin fragment, an antidiabetic drug. This paper introduces a novel precursor class, and time will tell whether selenonium compounds will be integrated into the radiochemist’s toolbox for routine PET radiochemistry production.

## Neurodegeneration, where does it all begin?

By Jan Passchier

The apparent disconnect between amyloid plaque density and level of cognitive impairment is well known. Things look a little more promising for tau tracers, and potentially the emerging alpha-synuclein tracers, but questions about treatment relevance remain. New PET tracers to explore the biological causes of neurodegeneration and open up new opportunities for drug development are required. Histone deacetylase 6 (HDAC6) is an interesting target given its role in the clearance of misfolded proteins. Initial clinical studies using [^18^F]bavarostat showed promise but the use of this tracer has been limited due to its manufacturing complexity.

**Fig. 3:**
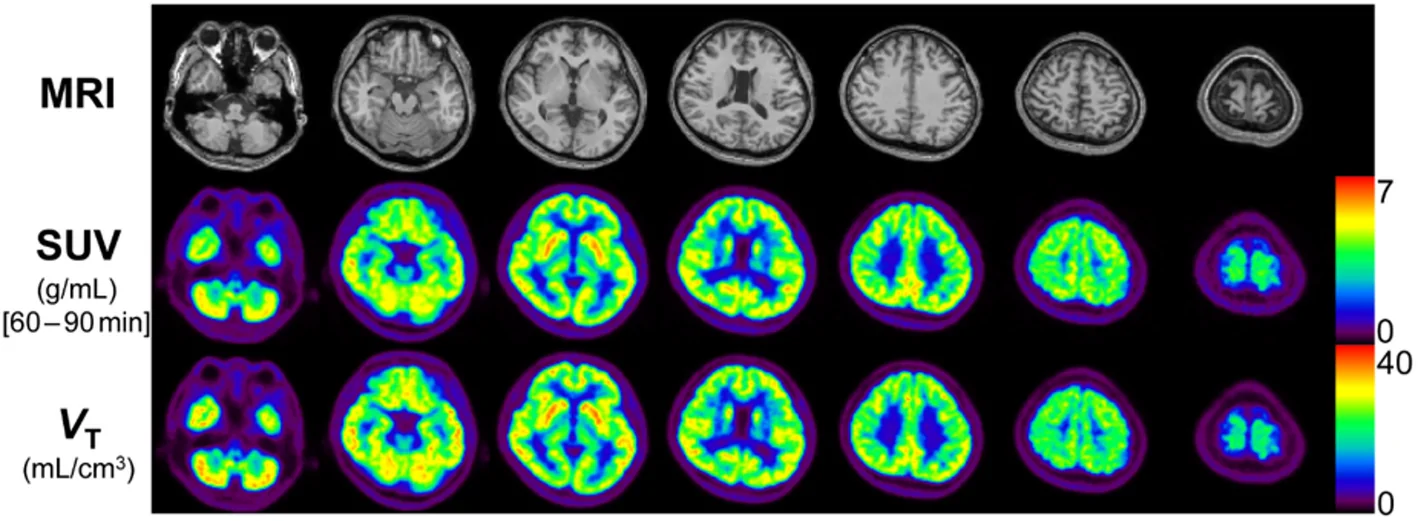
Representative brain MRI, [^18^F]FSW-100 SUV and Logan VT parametric maps. Image reproduced with permission (from Tago et al. 2026)

[^18^F]FSW-100 could be a more accessible alternative. In a recent paper by Tago and colleagues, the outcome of their comprehensive first-in-human study with [^18^F]FSW-100 was presented (Tago et al. 2026). In a cohort of healthy male volunteers, [^18^F]FSW-100 was well tolerated and showed good brain uptake (Fig. 3). Further work is underway to assess test/re-test variability in male and female cohorts as well as studies to confirm selectivity of this tracer over MBLAC2. If confirmed, this will provide an exciting opportunity to dive deeper into the pathogenesis of neurodegenerative disorders.

## ^18^F-Radiopharmaceutical diversification enabled by deaminative cross-electrophile couplings

By Giuseppe Carlucci

The highlighted paper introduces a versatile strategy for synthesizing ^18^F-labelled radiopharmaceutical analogues, which are widely used in positron emission tomography (PET) imaging and pharmaceutical research. Conventional ^18^F-labelling approaches typically require the preparation of a unique precursor for every analogue, making the exploration of structurally related tracers both time-consuming and labour-intensive. To overcome this limitation, a deaminative cross-electrophile coupling was adapted, a reaction that converts amine-derived compounds into intermediates for carbon-carbon bond formation, for application in radiochemistry (Ogilvy et al. 2026).

Their approach employs nickel-catalysed coupling between ^18^F-labelled fluoro(hetero)aryl iodides and alkyl 2,4,6-triphenylpyridinium salts prepared from primary or secondary amines. In practical terms, this enables a broad range of carbon-containing fragments to be introduced onto an already ^18^F-labelled aromatic scaffold, allowing rapid diversification of radiotracers at a late stage of synthesis. The method demonstrates broad functional-group tolerance and compatibility with drug-like molecules, delivering radiochemical conversions of up to 86%.

To highlight its utility, the authors applied the strategy to the late-stage diversification of a glycogen synthase kinase-3 (GSK-3) inhibitor, generating six distinct ^18^F-labelled analogues from a single common precursor. They further optimized the reaction through high-throughput ligand screening and demonstrated the scalability of the method by producing one analogue at giga-becquerel levels using semi-automated and fully-automated two-step protocols across three commercial radiosynthesis platforms.

Overall, this work represents a significant advance in ^18^F radiochemistry by enabling library-style synthesis of PET tracer analogues from a shared precursor. By simplifying analogue generation while maintaining broad chemical scope and compatibility with automated production workflows, the method has the potential to accelerate PET radiotracer development and facilitate more efficient drug discovery studies.

## New peptide-drug conjugate for extended therapy in prostate cancer

By Mohammed Al-Qahtani

Peptides generally have lower immunogenicity than antibodies, thereby reducing the risk of immune-related side effects and improving suitability for repeated administration in receptor-targeted precision therapy. Additionally, peptide–drug conjugates (PDCs) are substantially smaller, facilitating deeper tissue penetration, particularly in poorly permeable solid tumours, compared with antibody–drug conjugates (ADCs). Similarly, PDCs overcome the instability of nucleic acid-based ligands and offer greater flexibility for structural optimization.

**Fig. 4:**
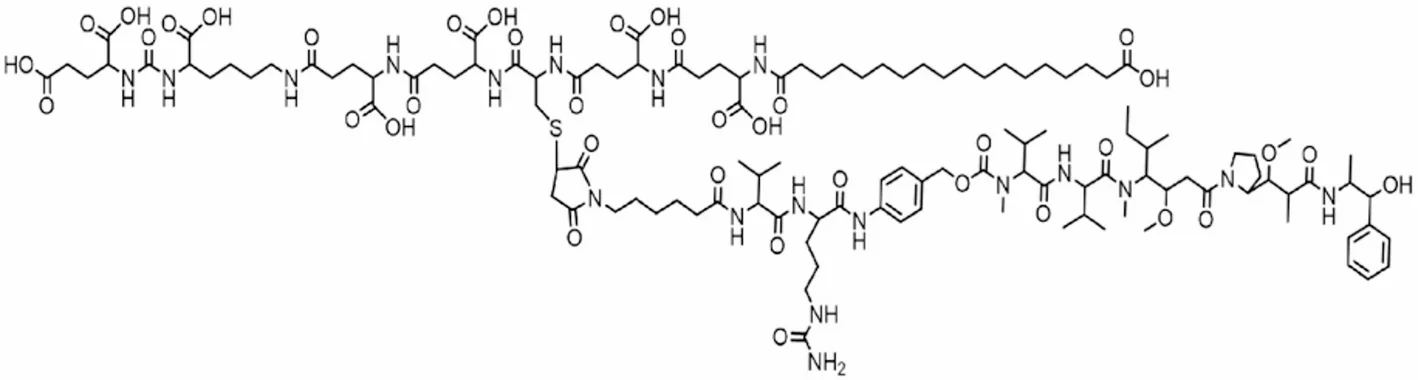
Scheme of stearic acid-modified PSMA-targeting peptide conjugated to MMAE via a linker, named PDC-C18 (With permission used from Qiu et al. 2026)

Thus, a series of PDCs was designed (Qiu et al. 2026), each incorporating various rationally selected functional moieties, starting with a Glu–Ureido–Lys ligand that targets prostate-specific membrane antigens (PSMA), the cytotoxic payload monomethyl auristatin E (MMAE), and a cathepsin B-cleavable linker composed of Valine–Citrulline (Val–Cit). Additionally, structural modifications involving exposure of free amines and subsequent acetylation of stearic acid are shown in Fig. 4. Among all candidates, the stearic acid-modified PSMA-targeting PDC (PDC-C18) stands out as a promising extended treatment for prostate cancer, as it rapidly forms stable nanocomplexes with human serum albumin (HSA) by stabilizing its hydrophobic interactions while maintaining its specificity for PSMA.

PDC-C18 demonstrates > 160-fold longer half-life, a 1.92-fold increase in mean residence time compared to PIBA-modified PDCs, and a 60-fold reduction in clearance. These extended-therapy properties result in an impressive 96.91% tumour suppression rate and avoid the side effects typically associated with traditional PIBA. Overall, the study offers an effective method to enhance the pharmacokinetics of PDCs and provides valuable insights for developing next-generation tumour-targeted precision therapies for prostate cancer.

## [^198^Au]Auranofin: an Example of a Non-receptor, High-capacity Site Theranostic

By Adriano Duatti

The design of metal-based therapeutic radiopharmaceuticals is dominated by the ubiquitous use of bifunctional ligands, consisting of a metal-chelating group and a bioactive moiety capable of selectively recognizing specific receptors expressed on the surface of tumour cells. For a given receptor, the copy number ranges approximately from 10⁴ to 10⁵ per cell. If each specific receptor can bind and internalize only a single therapeutic radiometal, it is difficult to accumulate a cytotoxic dose sufficient to kill the tumour cell; such receptors are classified as low-capacity sites. It can be hypothesized that high-capacity sites—which do not function exclusively through specific receptor-target interactions and are therefore not limited in radiometal accumulation—might be more efficient at delivering the necessary cytotoxic dose. A potential representative example is provided below (Bhardwaj et al. 2025).

The gold isotope ¹⁹⁸Au emits high-energy β⁻ particles with E_β_-_max_ = 0.96 MeV and t₁_/_₂ = 2.7 days, and 411.8 keV γ-rays (95.5%), nuclear characteristics that make it suitable for a theranostic. Auranofin is a gold compound originally approved for the treatment of rheumatoid arthritis. Structurally, auranofin is a lipophilic compound composed of a triethylphosphine ligand and a tetra-acetylated thioglucose group. Within the body, it undergoes deacetylation and ligand exchange within cells, releasing active gold(I) species and gold-thiolate complexes. Auranofin enters cells by displacing its original sugar ligand through exchange with membrane-bound thiol groups. Extracellular proteins and cysteine can also bind the gold atom. Once internalized, the gold moves progressively from the plasma membrane to the cytoplasm. This cellular trafficking contributes to the distribution of radioactive [^198^Au]auranofin within the tumour cell and, potentially, to the deposition of a cytotoxic dose. In summary, there are likely numerous molecular mechanisms—extending beyond the paradigm of specific receptor targeting—that could be exploited for radionuclide therapy, provided we can cast aside our dogmatic vision.

## Scaling the production of bromine-76 and bromine-77 labelled poly-ADP-Ribose-polymerase-targeted theranostics for preclinical use

By Bart Cornelissen

Auger electron therapy with ^77^Br, a theranostic pair with the positron-emitting ^76^Br holds great potential. The highlighted paper reports on an upscaled approach for the production of both radionuclides, to produce a radiolabelled PARP inhibitor (Johnson et al. 2026). Using enriched cobalt selenide targets, dry distillation, and workup, the authors were able to produce bromine isotopes robustly. Cu-mediated radiolabelling of a Bpin precursor progressed with excellent yields, similar to ^123^I or ^125^I reactions. Moreover, the authors had already shown the possible application of the Auger-electron emitting PARP inhibitor, which targets the short-range yet high linear energy transfer radiation from ^77^Br Auger electrons to tumour cell DNA, thereby irradiating them from the inside.

This report highlights the continued interplay between radiochemistry engineering, novel radionuclide production methods and careful optimisation, and improvement of radiochemistry and radiopharmaceutical vector design. The optimisation of bromide radionuclide production nearly completes the set of radiohalogens available for radiochemistry, with the exception of Cl (which only have either very short, or exceedingly long half-lives). This will allow careful dissection and comparison of ion size, and radiation types, especially for targeted radionuclide therapy (Bowden et al. 2021; Zheng et al. 2026; Muzzioli et al. 2026). In addition, this work highlights the increasing interest in Auger electron-emitting radiopharmaceuticals for targeted radionuclide therapy, as indicated by the increasing number of publications, also in this journal (Chu et al. 2026; Grundmane et al. 2026). Although production of Br radionuclides is currently limited to few sites, the relatively long half-lives (57 h for ^77^Br) allow long-distance shipping.

## Imaging KRAS-G12C mutations with PET: a novel approach for patient selection in targeted cancer therapy?

By Antonia Denkova

KRAS (Kirsten rat sarcoma) mutations play an important role in the development and progression of several cancer types, including non-small cell lung cancer (NSCLC) (Prior et al. 2020). Among these alterations, single-base missense mutations at codon 12 (G12) are among the most often occurring KRAS mutations identified in NSCLC, with the G12C variant representing a particularly important therapeutic target (Kwan et al. 2022). Adagrasib, a covalent KRAS-G12C inhibitor, has demonstrated promising clinical results, however, not all patients respond to this treatment requiring careful patient selection. Currently, the identification of patients who may benefit from KRAS-G12C-targeted therapies relies primarily on tumour biopsies. However, this approach can be challenging due to tumour heterogeneity and the limited ability of a single biopsy to represent the molecular profile of the entire tumour burden. To overcome these limitations, Willmann et al. developed a ^18^F-labelled KRAS-G12C inhibitor that enables PET imaging as a potential non-invasive tool for patient selection (Willmann et al. 2026). The authors designed three PET tracers based on the core pharmacophore of adagrasib by introducing ^18^F-labeled ethylene and oligoethylene glycol linkers onto the pyrrolidine ring. These modifications were aimed at improving aqueous solubility and optimizing the pharmacokinetic properties of the tracers. Among the evaluated compounds, [^18^F]KRAS490 demonstrated the most favourable profile, showing sufficient and selective tumour accumulation, as confirmed by blocking studies. The improved performance of this tracer was attributed to its reduced lipophilicity resulting from incorporation of a more polar polyethylene glycol linker compared with the other investigated molecules.

Furthermore, [^18^F]KRAS490 demonstrated the ability to cross the blood–brain barrier, exhibiting measurable brain uptake that was reduced upon blocking with non-radioactive inhibitor. These findings suggest that [^18^F]KRAS490 may also have potential application in visualizing KRAS-G12C alterations in the central nervous system.

## [^177^Lu]Lu-PSMA-617 in combination with pembrolizumab for treatment of metastatic castration resistant prostate cancer (PRINCE): a single-arm, phase 1b/2 study

By Jeroen Hendrikx

Since the introduction of [^177^Lu]Lu-PSMA-617 for treatment of prostate cancer in clinical practice, additional steps have been made to optimize treatment outcomes. In addition to radioligand therapy (RLT) monotherapy, combination therapies are also being considered. Recently, the results of the PRINCE trial were reported (Sandhu et al. 2026). In this single-arm, phase 1b/2 study, the co-administration of [^177^Lu]Lu-PSMA-617 and the immune checkpoint inhibitor (ICI) pembrolizumab was investigated. The combination was considered safe and a PSA50 response rate of 76% was observed (defined as PSA decline of ≥ 50% from baseline). Secondary endpoints included PSA-progression-free survival (result: 8.2 months; 95%CI 5.1–11.2), radiographic progression-free survival (result: 11.2 months; 95%CI 5.1–14.1) and overall survival (result: 20.8 months; 95%CI 13.5-not estimable). Although these survival rates have to be considered carefully because of the design of the study and number of patients, these results were comparable to VISION and TheraP trials. Albeit the mechanistic rationale for combination of RLT and ICIs is clear, the clinical impact of addition of pembrolizumab to RLT therapy in this trial seems of limited value compared to previous RLT monotherapy trials. Given that both RLT and ICI treatment are expensive options, further optimization is warranted to increase cost-benefit and make it clinically applicable. Since it is suggested that CD8 + T-cells need to be present and activated prior to RLT administration to enhance RLT outcomes (Shim et al. 2026), more mechanistic insights in (radio)biology and clinical insights in effects of dose timing will help to improve development of RLT combination therapies.

## The forgotten component of radiopharmaceuticals: why linkers deserve the spotlight

By Yann Seimbille

Current literature on radiopharmaceutical’s design is mainly emphasizing on the targeting vector, radionuclide, and chelator. The linker, one of the four major components of radiopharmaceuticals connecting the vector and the labeling moiety, is often neglected. This is why a review paper focuses on the critical role of the linker in the pharmacokinetic performance of radiopharmaceuticals (Stoddard et al. 2026).

The review is organized into two major sections. The first covers “static linkers”, designed to remain intact throughout the lifespan of the radiopharmaceuticals. These include the most widely used PEG-based linkers, and alternatives such as piperazine-, zwitterionic-, amino acid- and squaramide-based linkers, each offering distinct effects on hydrophilicity, rigidity, and pharmacokinetics. A dedicated subsection addresses albumin-binding linkers (e.g., maleimides, palmitic acid, ibuprofen, IPBA, Evans Blue, and albumin-binding domains), which extend circulation time by exploiting serum albumin’s long half-life. The second section addresses “dynamic linkers”, designed to release their payload in vivo. It includes linkers where cleavage is triggered by endogenous stimuli such as enzymes (e.g., renal brush border enzymes, cathepsins, caspases, β-glucuronidase, legumain) and non-enzymatic mechanisms (e.g., redox potential, acidity, and reactive oxygen species), or by exogenous stimuli (e.g., bioorthogonal click chemistry, NIR light/X-rays). The authors note that cleavable linkers are commonly used in antibody-drug conjugates, but they are far less prevalent in radiopharmaceuticals since decoupling of the radionuclide from its vector is not intrinsically necessary for efficacy.

The authors conclude by highlighting promising emerging technologies (zwitterionic linkers, IPBA-based albumin binding, click-to-release strategies), call for more rigorous comparative preclinical studies, and hope for more clinical validation of novel linkers.

## β-Hairpin conformation-oriented peptide evolution enables high-affinity PD-L1 radiopharmaceuticals

By Xing Yang

Peptide-based radiopharmaceuticals have emerged as an attractive platform for molecular imaging and targeted radionuclide therapy, particularly for targeting protein-protein interactions (PPIs). However, most peptide ligands identified by display technologies remain conformationally flexible, resulting in suboptimal target affinity, rapid proteolytic degradation, and unfavourable pharmacokinetics.

To address this challenge, Liu and co-workers developed a β-hairpin conformation-oriented evolution strategy using programmed death ligand 1 (PD-L1) as a model β-sheet-mediated PPI target (Liu et al. 2026). Starting from the PD-L1-targeting lead peptide TPP-1, the authors progressively stabilized the β-hairpin architecture through sequential β-turn optimization, incorporation of a Trpzip motif, and macrocyclization, ultimately generating the conformationally locked cyclic peptide TPP-10. The engineered cyclic peptide exhibited picomolar affinity for PD-L1 (K_D_ = 6.24 × 10^− 3^ nM), with a nearly 600-fold improvement over TPP-1, together with markedly enhanced in vivo stability. Complementary structural analyses, including circular dichroism (CD), Fourier-transform infrared (FTIR), nuclear magnetic resonance (NMR), X-ray diffraction (XRD), small-angle X-ray scattering (SAXS), and molecular dynamics simulations validated that the engineered modifications effectively stabilized the desired β-hairpin conformation. Radiolabelling of TPP-10 with both ^68^Ga and the longer-lived radionuclide ^64^Cu further demonstrated excellent tumour-targeting capability and PET imaging performance in multiple tumour models.

Beyond the development of a high-performance PD-L1 PET radiotracer, the broader significance of this study lies in highlighting β-hairpin conformation-oriented engineering as a promising strategy for the design of peptide-based radiopharmaceuticals. By showing that rational stabilization of the β-hairpin architecture can improve both target recognition and pharmacokinetic properties, this work provides a rational design framework for developing peptide radiopharmaceuticals against challenging β-sheet-mediated PPI.

## The brain’s wiring constrains synaptic loss in alzheimer’s disease

By Hongmei Jia

Synaptic loss is one of the strongest biological correlates of cognitive impairment in Alzheimer’s disease (AD), but why it follows a characteristic spatial pattern across the brain remains incompletely understood. In a *Nature Communications* study, researchers from Fudan University and collaborating institutions investigated whether the brain’s intrinsic network architecture helps shape where synaptic degeneration occurs (Luan et al. 2025).

The study combined SV2A PET imaging, an in vivo marker of synaptic density, from amyloid-positive AD participants and amyloid-negative controls with normative functional and structural connectome data. Brain regions with stronger connectivity showed more similar levels of synaptic loss. Moreover, synaptic loss in a given region was related to connectivity-weighted synaptic loss in its connected neighbours, indicating that regional synaptic vulnerability is partly constrained by the brain’s network topology.

Using an epicenter-based model, the authors further demonstrated that regions more strongly connected to putative synaptic loss epicenters exhibited greater cross-sectional synaptic loss and faster longitudinal decline. Importantly, these network-based patterns were observed at both group and individual levels and accounted for heterogeneous spatial subtypes of synaptic degeneration. Plasma phosphorylated tau181 was associated with stronger connectivity-mediated synaptic loss, while post-mortem tissue analyses confirmed reduced SV2A expression in tau-rich regions, supporting a modulatory role of tau pathology.

Together, these findings suggest that synaptic degeneration in AD is not simply determined by local pathological burden, but follows a network-defined vulnerability landscape shaped by brain connectivity and modulated by phosphorylated tau. This network-based framework may provide new opportunities to predict individual patterns of synaptic vulnerability and subsequent synaptic decline based on network features, thereby informing the development of imaging biomarkers for disease progression.

## [^68^Ga/^177^Lu]-Labelled TSPO ligands for imaging and therapy of glioblastoma

By Ming-Rong Zhang

The 18 kDa translocator protein (TSPO), mainly located on the outer mitochondrial membranes, is overexpressed in many malignant tumours, such as brain cancer, lung cancer, breast cancer, pancreatic cancer and prostate cancer. Increased TSPO levels are closely related to high-grade cancers and poor patient prognosis, making it an attractive target for cancer diagnosis and radiotherapy. A series of TSPO-targeting radioligands was developed for PET imaging and radiotherapy of glioblastoma (Wang et al. 2026). Four novel radioligands were designed and synthesized based on the TSPO PET ligand [^18^F]FEMPA in clinical use, enabling simultaneous chelation with PET and therapeutic radionuclides. They conjugated the FEMPA scaffold with DOTA *via* PEG linkers of different lengths to generate four new compounds TSPO-1 ~ 4. These compounds were labelled with Ga-68 to yield [^68^Ga]TSPO-1 ~ 4 for in vitro and in vivo evaluations. PET with [^68^Ga]TSPO-1 ~ 4 detected TSPO expression in orthotopic U87MG tumours, outperforming [^18^F]FDG in terms of specificity and tumour-to-brain ratio. Among them, [^68^Ga]TSPO-1 showed the highest uptake of radioactivity in orthotopic U87MG tumours while displaying fast clearance compared to other three radioligands. Further, [^177^Lu]TSPO-1 significantly inhibited U87MG tumour growth in a mouse model with favourable biosafety. It was noted that the four radioligands developed in this study showed low to modest BBB penetrating ability, so their potential should be limited to the PET imaging and radiotherapy of glioblastoma with a disrupted BBB.

## Toward truly matched antibody radiotheranostics

By Min Yang

**Fig. 5:**
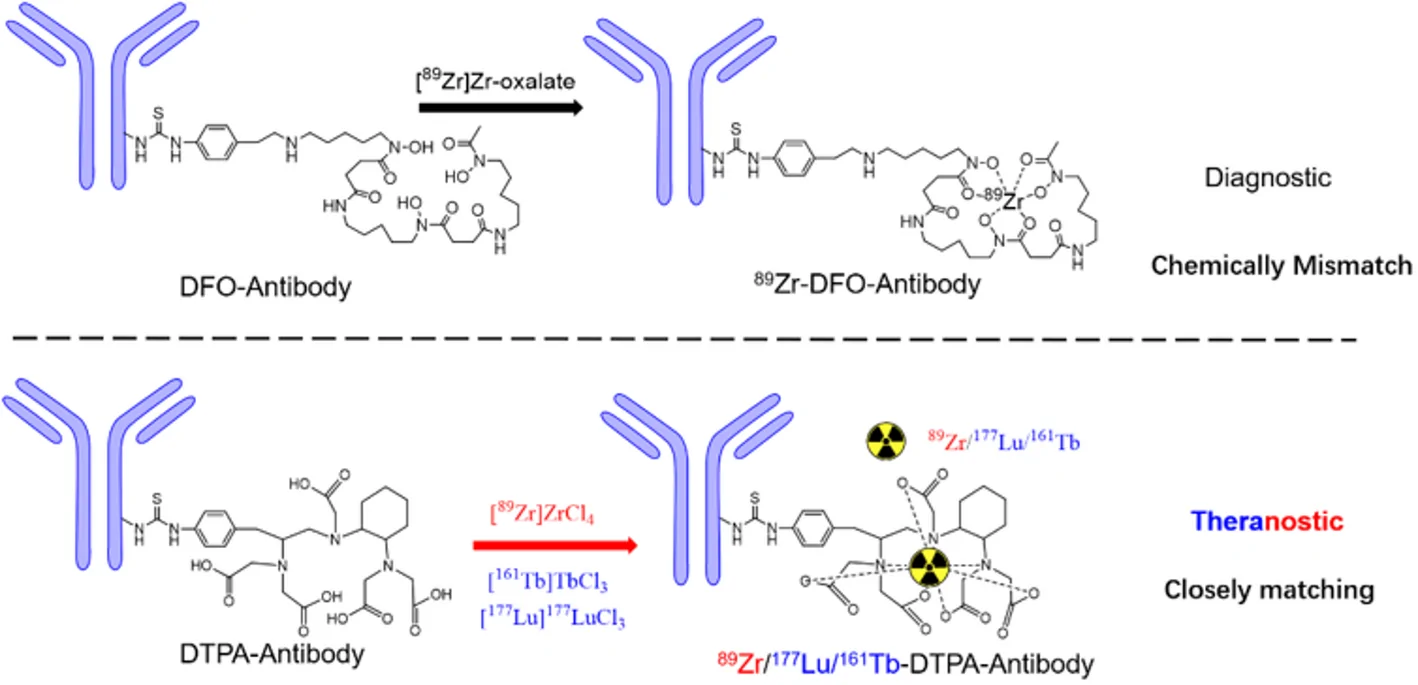
Chelator-matched ⁸⁹Zr/¹⁷⁷Lu/¹⁶¹Tb radiotheranostic pairs using CHX-A″-DTPA overcome the conventional DFO-vs-DTPA chemical mismatch, enabling imaging agents that more accurately predict therapeutic biodistribution and dosimetry

Antibody-based radiotheranostics is an important component of precision oncology, yet a chemical inconsistency remains. Most ^89^Zr-immunoPET agents use deferoxamine (DFO), whereas therapeutic radioimmunoconjugates often employ DTPA- or DOTA-based chelators for ^177^Lu and ^161^Tb. Diagnostic and therapeutic agents may therefore share the same antibody and target while differing in chelator chemistry, potentially reducing the accuracy of imaging-based biodistribution and dosimetry predictions. Prior attempts to label DTPA–antibody conjugates with ⁸⁹Zr-oxalate were unsuccessful, likely because DTPA cannot efficiently transchelate Zr⁴⁺ from the stable oxalate complex. Lyashchenko and colleagues (Lyashchenko et al. 2026) overcame this barrier using oxalate-free ^89^ZrCl₄, enabling direct labelling of CHX-A″-DTPA–antibody conjugates and construction of chelator-matched ^89^Zr/^177^Lu/^161^Tb radiotheranostic pairs (Fig. 5). The radiolabelling reaction requires only 10 min, accommodates activities up to 3.4 GBq, and preserves radioimmunoreactivity while maintaining high radiochemical purity and antibody binding. Compared with ^89^Zr-DFO-pertuzumab, the DTPA analogue produced nearly tenfold fewer low-molecular-weight radioactive species after 48 h and retained approximately 1.2-fold more radiolabelled monomer in pooled human serum after 120 h. In the BT-474 xenograft model, it showed lower hepatic uptake, higher blood and tumour uptake, and a biodistribution more closely matching ^161^Tb-DTPA-pertuzumab. By matching the antibody–chelator platform rather than only the antibody and target, this work moves antibody radiotheranostics toward more complete chemical matching. The resulting imaging agent may serve as a more representative surrogate for radioimmunotherapy, potentially improving patient selection, treatment planning, and individualized dosimetry. Although broader validation across additional antibody systems and clinical studies is still needed, this study establishes a foundational framework for chemically matched antibody radiotheranostics.

## The european horizon of radiopharmaceuticals

By Lars Perk

For this Highlight Commentary, I did not select a scientific publication but a recent European Medicines Agency (EMA) horizon-scanning report on radiopharmaceuticals (Radiopharmaceuticals EU-IN Horizon Scanning Report, 2026). Reports from regulatory agencies, as well as patent literature, are sometimes overlooked by the scientific community. However, these sources can offer valuable insights into the future direction of research, development, and clinical translation. Ultimately, our shared goal is to bring innovative radiopharmaceuticals from the laboratory to the bedside.

The report highlights the rapid growth of radiopharmaceutical research and clinical trials. Currently, approximately 70 radiopharmaceuticals are approved by the U.S. Food and Drug Administration (FDA) and around 20 by the EMA, with 75–80% intended for diagnostic applications. Most products approaching marketing authorization are labeled with ^177^Lu, ^68^Ga, or ^18^F, but also with ^64^Cu, ^89^Zr, ^224^Ra, ^225^Ac, ^124^I, ^131^I, and ^99m^Tc.

The regulatory landscape is expected to evolve substantially in the coming years, with several important guidelines under revision or development, including EU GMP Annex 3 and the EMA guideline on the clinical evaluation of therapeutic radiopharmaceuticals in oncology. Key challenges remain the lack of harmonized implementation of regulations across Europe and radionuclide or product supply shortage or disruptions.

I encourage readers to explore such reports and engage in policy discussions that will shape the future of our field.

## MRI probe for bioorthogonally catalyzed activation imaging

By Peter Caravan

Activatable MRI probes that produce signal by changing the relaxation time of water often suffer from the limitation of still producing signal in their “off” state such that it is difficult to distinguish specific signal due to activation from nonspecific signal due to distribution. Guo and colleagues designed a low spin Fe(II) complex with near zero relaxivity such that it does not provide any MRI signal after intravenous administration (Guo et al. 2026). The authors also developed a biorthogonal ruthenium catalyst that could catalytically convert the low spin Fe(II) complex to high spin Fe(III) in aqueous media with a 200-fold turn on in relaxivity. The authors used pre-targeting to deliver the ruthenium catalyst to a tumour in a mouse model and then showed that intravenous administration of the low spin Fe(II) complex produced bright MRI signal specifically at the tumour because of oxidation by the ruthenium catalyst. This exceptionally innovative paper highlights the potential of this technology for background-free molecular imaging.

## Toward dosimetry-guided alpha therapy with paired lead radioisotopes

By Zhen Cheng

Targeted alpha-particle radionuclide therapy has attracted increasing attention in nuclear medicine because of several prominent advantages of alpha emitters including high linear energy transfer and short emission path length, which induce highly cytotoxic and difficult-to-repair DNA damage within targeted tumour cells. This physical profile not only makes alpha-emitting radiopharmaceuticals attractive for treatment of micro-metastatic disease or heterogeneous tumours, but also places stringent demands on target selectivity, pharmacokinetics, and dosimetry. As alpha-radiopharmaceuticals move toward broader clinical translation, selecting appropriate patients, visualizing treatment-relevant biodistribution, and estimating tumour and normal-organ absorbed doses have become increasingly important for optimizing therapeutic benefit while managing radiotoxicity.

A recent phase 0 imaging trial took an important step toward addressing these needs by evaluating [^203^Pb]Pb-VMT-α-NET as a SPECT/CT surrogate for treatment planning with [^212^Pb]Pb-VMT-α-NET in patients with refractory or relapsed metastatic neuroendocrine tumours (Graves et al. 2026). Because ^203^Pb and ^212^Pb are paired radioisotopes with imaging and therapy capability, respectively, their combination use offers a practical route to link pretherapeutic imaging to subsequent alpha-particle therapy. In the study [^203^Pb]Pb-VMT-α-NET demonstrated promising imaging performance for neuroendocrine tumour lesions larger than 1 cm, with a reported sensitivity of 94% in this size range. Quantitative SPECT/CT also enabled estimation of normal-organ and tumour dosimetry, supporting individualized treatment planning for [^212^Pb]Pb-VMT-α-NET therapy.

This work represents an important step toward pretherapeutic imaging- and dosimetry-guided personalized alpha therapy. Further studies in larger cohorts are needed to determine how accurately ^203^Pb-based imaging predicts therapeutic response and toxicity after [^212^Pb]Pb-VMT-α-NET therapy. The study provides a practical strategy for integrating Pb-based imaging into alpha-therapy planning.

## Imaging copper metabolism to guide therapy in triple-negative breast cancer

By Tamer Sakr

Triple-negative breast cancer (TNBC) represents one of the most challenging subtypes of breast cancer as it has an aggressive biological behaviour and limited treatment options. A recent study explored [^64^Cu]CuCl₂-PET/CT as a new radiopharmaceutical model to visualize copper metabolism (Lewis et al. 2026). [^64^Cu]CuCl₂-PET/CT showed preferential localization in TNBC that was confirmed by decreased radiotracer accumulation after copper chelation with tetrathiomolybdate (Hamsell et al. 2010). A valuable translational bridge toward clinical applications was provided in this study using different animal models starting from murine models to cats with naturally occurring mammary carcinoma (Adega et al. 2016). [^64^Cu]CuCl₂-PET/CT represents a dual action radiotracer that can act as a diagnostic imaging agent and monitor the biological responses to copper-targeted therapy (Lewis et al. 2026). This confirms that molecular imaging can play a potential role for the development and evaluation of novel strategies for aggressive breast cancer therapy.

## How much carrier is in your no-carrier-added radiochemical?

By Jan Rijn Zeevaart

In line with the fast-developing field of nuclear medicine, innovative new radioisotopes to be used as radiochemicals (starting material for radiopharmaceuticals) are being proposed. The area of Auger-emitting (AE) radioisotopes are attracting particular interest but it is common knowledge that the specific activity (molar activity) has to be as high as possible where receptor-targeting radiopharmaceuticals are envisaged. No-carrier-added (n.c.a) refers to a preparation of a radioactive isotope that is essentially free from stable isotopes of that same element, while carrier-free can only be achieved in rare cases, where the theoretical maximum specific or molar activity is unambiguously proven (Coenen et al. 2018).

The apparent success of ^161^Tb re-sparked interest in AE. One such AE radioisotope is Pd-103 which has been produced as a brachytherapy source for prostate cancer treatment over two decades now with a specific activity of ≥ 60 Ci/g (molar activity of 0.23 MBq/nmol). Essentially n.c.a. ^103^Pd is produced through irradiation of Rh foils and no other palladium isotopes are introduced in the process. However, higher molar activities can be obtained by irradiating high purity Rh for production of Pd-103 as demonstrated by the Engle group in Wisconsin a recent paper (Deville et al. 2026).

The production of n.c.a. Pd-103 within this study gave varying molar activities and comparing these values to what has been achieved for ^161^Tb (Gracheva et al. 2019) and commercial ^177^Lu can be seen in Table 1 below.

**Table 1:** A comparison of molar activities obtained for Pd-103, Lu-177 and Tb-161

Radioisotope	Theoretical Molar Activity (MBq/nmol)	Measured Molar activity (MBq/nmol)	% of the theoretical Molar Activity	Source
^177^Lu	728	648	89	NTP Radioisotopes CoA
^161^Tb	696	180	25.9	Gracheva et al. 2019
^103^Pd (from recycled Rh targets)	285	30	10.5	Deville et al. 2026
^103^Pd (from commercial Rh foil)	285	1.5	0.5	Deville et al. 2026
^103^Pd (from electroplated Rh targets	285	1.1	0.4	Deville et al. 2026

The value for ^177^Lu is higher than ^161^Tb (still in R&D phase), likely due to higher production activities which favour inclusion of smaller amounts of stable Lu. Despite good progress being made the values for ^103^Pd are lower as cyclotrons generally produce substantial lower activities per batch which makes it more difficult to achieve as high molar activities. The highest molar activity for ^103^Pd was achieved where recycled Rh was used because the Pd impurities would have been removed from the target material in the previous production process. Using starting material where the stable isotopes of Pd are removed from the Rh target material (prior to irradiation) is essential in reducing the carrier in the n.c.a. radiochemical.

Acknowledgement. Nick van der Meulen for discussion on the topic

## Locking the target: the rise of covalent radiopharmaceuticals in oncology

By Julien Leenhardt

**Fig. 6:**
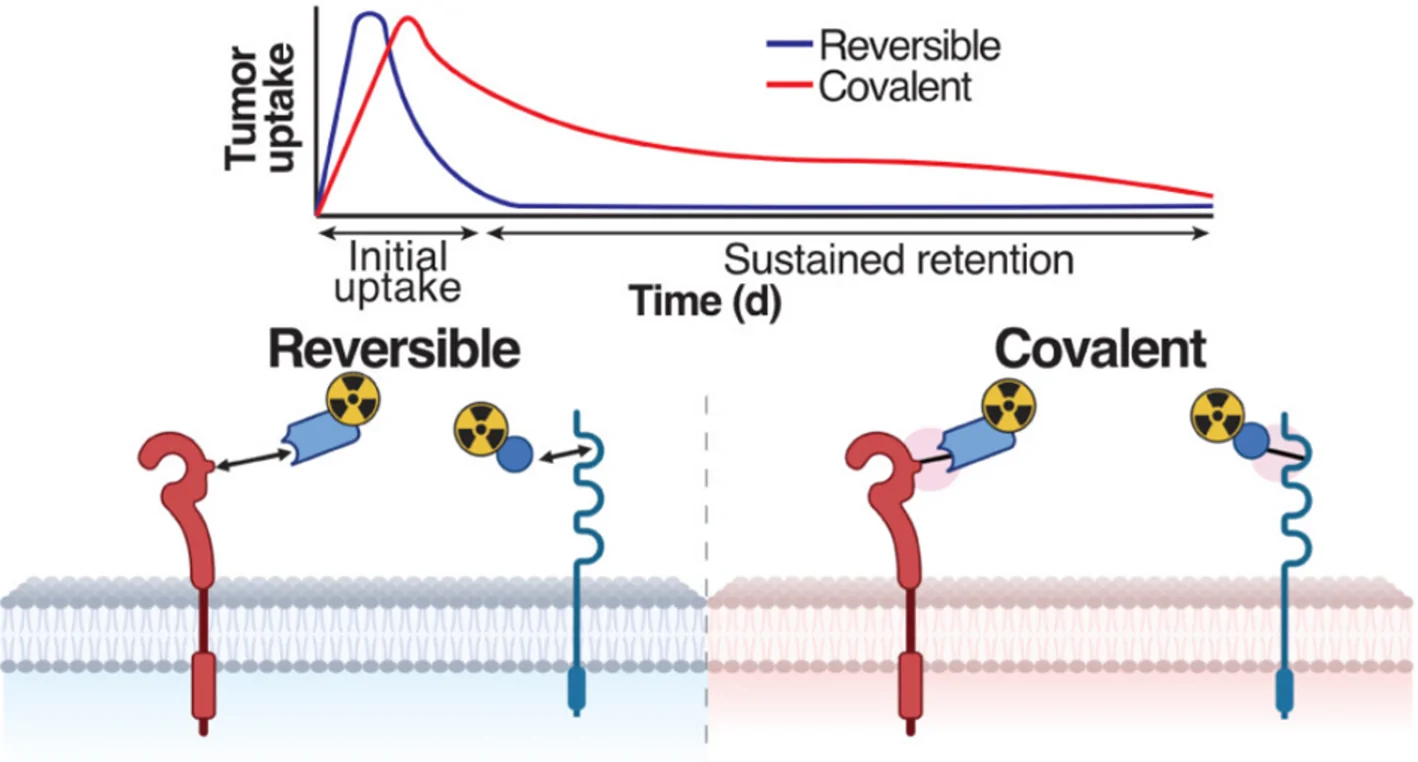
Concept of covalent versus reversible binding radiopharmaceuticals (with permission from Klauser et al., 2026)

Conventional radiopharmaceuticals rely on reversible target interactions inherently limited by dissociation, receptor recycling, and rapid clearance which undermine tumor retention, particularly for long-lived radionuclides. Covalent radiopharmaceuticals as an emerging strategy are discussed in a Perspective article (Klauser et al., 2026) to overcome these limitations by irreversibly anchoring radioligands to their molecular targets. The concept is deceptively simple: equip a radiopharmaceutical with a carefully positioned electrophilic warhead capable of reacting with a nearby nucleophilic residue only after target binding (Fig. 6).

The authors highlight a range of representative platforms, including KRAS G12C-targeting small molecules, HER2-targeted covalent nanobodies generated using PERx chemistry, redox-responsive tracers, and ketobenzothiazole-based peptidomimetics targeting the ST14 protease, illustrating the breadth of this emerging field. A particularly compelling example is the work of Cui et al. (*Nature*, 2024), who reported a SuFEx-engineered FAP inhibitor achieving proximity-enabled covalent ligation to a tyrosine residue, resulting in markedly enhanced tumor uptake and retention over the parent tracer in mice, with a pilot clinical study also showing improved lesion detection over conventional FAPI.

Beyond academia, interest in covalent radiopharmaceuticals is also gaining momentum in the biotechnology sector, with companies such as ActiThera and Enlaza Therapeutics advancing covalent conjugation platforms for both diagnostic and therapeutic applications.

The field nevertheless faces significant challenges, including controlling off-target reactivity, demonstrating long-term safety and establishing an appropriate regulatory framework. Despite these hurdles, the ability to selectively lock radiopharmaceuticals onto their targets could represent a paradigm shift in nuclear medicine, paving the way for a new generation of radiotheranostic agents.

## A novel PET strategy to image the procoagulant state in alzheimer’s disease

By Juan Pellico

**Fig. 7:**
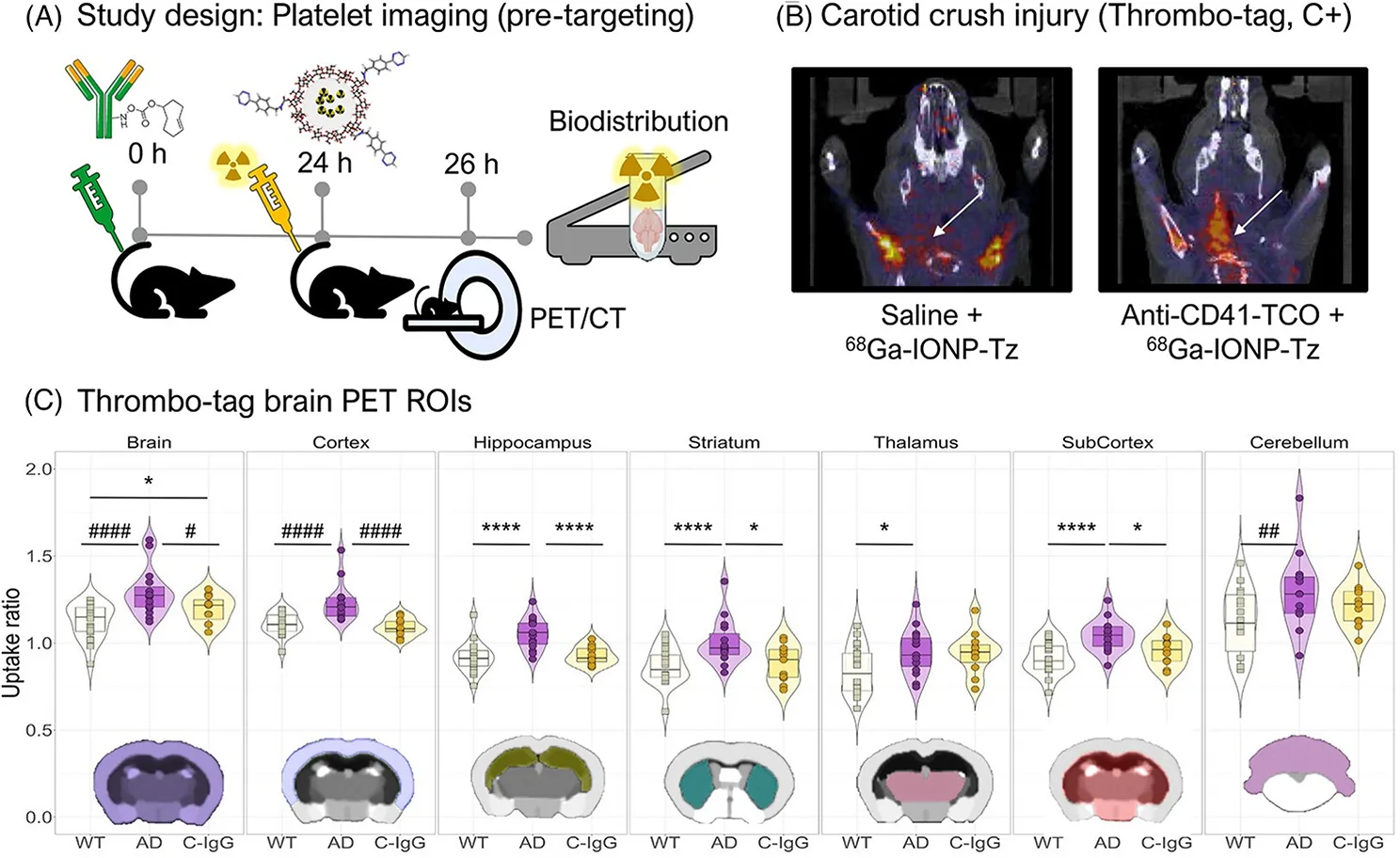
Thrombo-tag (i.e., anti–CD41-TCO + [^68^Ga]Ga-IONP-TZ) enables in vivo cerebral detection of platelet accumulation in TgCRND8 AD mice. (**A**) Study design. (**B**) Representative PET images of the carotid crush injury model (C+) showing high radiotracer uptake in the injured carotid region of WT mice injected with the anti–CD41-TCO + [^68^Ga]Ga-IONP-TZ. (C) PET ROI analyses across brain regions comparing AD versus WT mice (t-test: *p < 0.05, ****p < 0.001; Mann–Whitney: #*p* < 0.05, ##*p* < 0.01, ####*p* < 0.001). Adapted with permission from [Alzheimer’s Dement. 2026;22:e71652] under terms of the Creative Commons Attribution-NonCommercial-NoDerivs License

A novel PET imaging strategy has been reported to detect the procoagulant state associated with Alzheimer’s disease (AD) by targeting fibrin and platelets, two key components of cerebral microthrombi (Ceron et al. 2026). The authors first developed a directly labelled fibrin PET probe, using a commercial fibrin-binding peptide functionalised with deferoxamine (DFO) and radiolabelled with ^89^Zr. The long half-life of ^89^Zr (78.4 h) enabled longitudinal imaging of the same TgCRND8 AD and control mice over five consecutive days, allowing optimization of imaging time points and demonstrating that 24 h post-injection provided the highest contrast between AD and control mice. To improve clinical translation, the study then introduces a bioorthogonal pre-targeting strategy based on the fast tetrazine–trans-cyclooctene (Tz–TCO) click reaction. Here, the fibrin-binding peptide was first modified with TCO and administered one day before imaging, allowing clearance of unbound probe. Subsequently, a ^68^Ga-labelled DOTA-tetrazine was injected shortly before PET acquisition, producing in vivo radiolabelling only at the target site. This approach combines the favourable dosimetry and availability of ^68^Ga with improved target-to-background ratios while maintaining high target specificity. The most innovative radiochemical contribution is the application of the platelet-targeted “Thrombo-tag” probe, which couples an anti-CD41 antibody functionalised with TCO to ^68^Ga-labelled iron oxide nanoparticles bearing tetrazine (Fig. 7). Unlike conventional small-molecule radiotracers, this nanoradiotracer exploits both in vivo click chemistry and nanoparticle-mediated signal amplification, providing greater target engagement and improved sensitivity for detecting platelet accumulation associated with cerebral microthrombi.

Overall, the authors applied elegant (radio)chemistries providing the first in vivo neuroimaging approach to detect the procoagulant state associated with AD. Their findings suggest that this PET imaging strategy can potentially become a valuable clinical tool to identify AD patients with a procoagulant phenotype after further validation, enabling patient stratification and personalized therapies, such as antiplatelet or fibrin-targeted treatments.

## Data Availability

Datasets mentioned in this article can be found in the cited articles.

## Abbreviations

AD: Alzheimer’s disease
AE: Auger emitter
CD: Circular dichroism
DFO: Deferoxamine
DNA: Deoxyribonucleic acid
EMA: European medicines agency
FAPI: Fibroblast activating protein inhibitor
FDA US: Food and drug administration
FDG: Fludeoxyglucose
FTIR: Fourier transformation infrared
KRAS: Kirsten rat sarcoma
n.c.a: non carrier added
NMR: Nuclear magnetic resonance
PDC: Peptide-drug conjugate
PD-L1: Programmed death ligand 1
PPI: Protein-protein interaction
PET: Positron emission tomography
PSMA: Prostate specific membrane antigen
SAXS: Small angle X-ray scattering
SPECT: Single photon emission computed tomography
TCO: Trans-cyclo-octene
TSPO: Translocator protein
XRD: X-ray diffraction
